# Supplementary material for: WordCommentsAnalyzer: A windows software tool for qualitative research
Source: F1000Res. 2018 Sep 4;7:536. Originally published 2018 May 3. [Version 2] doi: 10.12688/f1000research.14819.2 (PMC6137411; doi:10.12688/f1000research.14819.2)
Supplement: Supplementary file 3 [file f1000research-7-17614-s0002.tgz › b2a79496-4ada-47c4-9682-a99ce867836d.zip!/Eight Iranian Health Journals/Journal of Research in Rehabilitation Sciences _15.docx]

© Journal of Research in Rehabilitation Sciences . This work is licensed under CC BY-NC 4.0 (https://creativecommons.org/licenses/by-nc/4.0/).

Volume & Issue: دوره 11، شماره 2: 1394

Title: تأثیر پنجه ساچ و داینامیک بر متغیرهای کینتیکی جذب شوک، نیروی برآیند عکس‌العمل زمین و ایمپالس عمودی در فاز استانس دویدن افراد قطع عضو یک‌طرفه ترانس‌تیبیال

http://jrrs.mui.ac.ir/index.php/jrrs/article/view/2044

مقدمه: افراد معلول دچار قطع عضو ترانس‌تیبیال هنگام دریافت اندام مصنوعی، ناگزیر به استفاده از یک نمونه از پنجه‌ها، جهت جایگزینی عضو از دست رفته هستند. ویژگی‌های بیومکانیکی دویدن افراد قطع عضو ترانس‌تیبیال با پنجه‌های مختلف مورد بررسی قرار گرفته است. علیرغم اینکه پنجه ساچ (SACH) و داینامیک شرکت (Otto Bock) جزء رایج‌ترین پنجه‌های مصرفی در ایران است، بررسی عملکرد این پنجه‌ها در فعالیت‌های حرکتی کمتر مورد توجه قرار گرفته است. هدف از این پژوهش بررسی تأثیر پنجه ساچ و داینامیک بر برخی متغیرهای کینتیکی در فاز استانس دویدن افراد قطع عضو یکطرفه ترانس‌تیبیال می‌باشد. مواد و روش‌ها: 8 مرد قطع عضو یکطرفه ترانس‌تیبیال در این مطالعه شرکت کردند. هر آزمودنی با هر یک از پنجه‌ها سه بار مسیر 12 متری را با سرعت 5/2 متر بر ثانیه می‌دوید. از صفحه نیرو Kistler برای جمع آوری اطلاعات کینتیکی و از سیستم آنالیز حرکت Vicon برای کنترل سرعت دویدن استفاده شد. برای تعیین اثر پنجه‌ها از آزمون آماری آنالیز واریانس با اندازه‌های تکراری در داده‌های نرمال و آزمون فریدمن در داده‌های غیر نرمال استفاده شد (05/0P < ). یافته‌ها: پای قطع عضو افراد قطع عضو ترانس‌تیبیال یکطرفه شاخص جذب شوک (قلّه‌ی نیروی عمودی عکس‌العمل زمین هنگام تماس پاشنه) نداشت. نیروی برآیند عکس‌العمل زمین میان پنجه ساچ، داینامیک و پنجه مورد استفاده آزمودنی‌ها به طور معناداری متفاوت بود (001/0P < ) که پنجه ساچ نیروی برآیند عکس‌العمل زمین بیش‌تری (001/0P < ) نسبت به پنجه داینامیک و پنجه اصلی داشت اما ایمپالس عمودی میان پنجه‌ها تفاوت معناداری نداشت (38/0P = ). نتیجه‎گیری: نتایج مطالعه حاضر نشان داد که هنگام دویدن، نیروی برآیند عکس‌العمل زمین در پای قطع عضو با پنجه ساچ بیش‌تر از پنجه داینامیک است و می‌تواند موجب افزایش خطر ابتلا به درد پتلوفمورال در پای قطع عضو شود.

Title: بررسی و مقایسه رشد زبانی کودکان کاشت حلزون‌شده و کودکان دارای شنوایی طبیعی

http://jrrs.mui.ac.ir/index.php/jrrs/article/view/2087

مقدمه: ناشنوایی اثرات نامطلوبی بر رشد زبان کودک خواهد داشت. امروزه، انجام عمل جراحی کاشت حلزون فرصتی برای رشد و تحول زبان در کودکان ناشنوا فراهم آورده است. پژوهش حاضر با هدف بررسی رشد زبانی کودکان نا‌شنوا بعد از گذشت دو سال از عمل جراحی کاشت حلزون و مقایسه آن‌ها با کودکان دارای شنوایی طبیعی انجام شده است. مواد و روش‌ها: جامعه آماري کودکان کاشت حلزون ‌شده شامل کلیه کودکانی بودند که در بیمارستان الزهرای (س) اصفهان، تحت عمل جراحی کاشت حلزون قرار گرفته بودند. از این جامعه 35 کودک (18 پسر و 17 دختر) که حداقل دو سال از عمل جراحی آن‌ها گذشته بود، به روش نمونه‌گیری تصادفی انتخاب و مورد بررسی قرار‌ گرفتند. در گروه کودکان دارای شنوایی طبیعی، جامعة آماري شامل کليه کودکانی بودند که در مراکز پیش‌دبستانی و دبستان شهر اصفهان، تحت آموزش قرار گرفته بودند. از این جامعه، 35 کودک (18 پسر و 17 دختر) به شیوه نمونه‌گیری خوشه‌ای تصادفی چند مرحله‌ای انتخاب شدند. داده‌های پژوهش با استفاده از مقیاس رشد و تحول زبان جمع‎‌آوری شد و در پایان داده‌های آماری از طریق روش همبستگی و تحلیل کواریانس، مورد بررسی قرار گرفتند. یافته‌ها: اگر چه کودکان کاشت حلزون‌شده نمره‌های بالایی در مقیاس رشد زبان دریافت می‌کنند، با این وجود بین ميانگين امتيازات  رشد زبان در دو گروه تفاوت معنی‌داری وجود داشت(001/0 ≥ P). همچنین بین سن و رشد زبان رابطه مثبت معنی‌داری وجود داشت (010/0 ≥ P، 491/0 = r). نتیجه‎گیری: با توجه به یافته‌های پژوهش حاضر می‌توان نتیجه گرفت که اگر چه انجام عمل جراحی کاشت حلزون موجب رشد زبان در کودکان ناشنوا می‌شود ولی نباید انتظار داشت که بعد از گذشت دو سال از عمل جراحی، کودکان کاشت حلزون شده در مهارت‌های زبانی مشابه کودکان دارای شنوایی طبیعی عمل نمایند.

Title: تعیین تأثیر شش هفته تمرین راه رفتن با پاهای رو به داخل در منزل بر عملکرد افراد مبتلا به آسیب لیگامان متقاطع قدامی در آزمون لی لی تک پا و آزمون 8 ـ مطالعه مقدماتی

http://jrrs.mui.ac.ir/index.php/jrrs/article/view/2294

مقدمه: آسيب ليگامان صليبي قدامي زانو شايع تر از ساير ليگامان هاي اين مفصل مي‌باشد. هدف اوليه توانبخشي بعد از آسيب ليگامان صليبي قدامي، بازگرداندن عملکرد مي‌باشد علي رغم تحقيقات موجود در مورد تغييرات پارامترهاي مختلف راه رفتن در حين Toe-in walking، تاکنون تأثير اين ورزش بر عملکرد بيماران مبتلا به آسيب ليگامان متقاطع قدامي به صورت باليني مورد بررسي قرار نگرفته است. بنابراين، مطالعه حاضر به عنوان يک مطالعه مقدماتي براي تعيين اثر شش هفته تمرين خانگي نظارت شده راه رفتن با پا هاي رو به داخل بر عملکرد افراد غيرکوپر دچار ضايعه ليگامان صليبي قدامي در آزمون لي لي تک پا و آزمون 8 طراحي شد. مواد و روش‌ها: 7 مرد جوان غيرکوپر مبتلا به آسيب ليگامنت متقاطع قدامي براساس معيارهاي معرفي شده توسط Fitzgerald انتخاب شدند. به بيماران آموزش داده شد که به مدت شش هفته، هفت روز هفته روزانه سي دقيقه با کفش با هر دو پا در حالت رو به داخل در منزل راه بروند. عملکرد افراد در آزمون لي لي تک پا و آزمون 8  قبل و بعد از مداخله بررسي شد. همچنين کليه شرکت کنندگان پرسشنامه  International Knee Documentation Committee(IKDC) را در ابتدا و پس از پايان دوره مداخله تکميل نمودند. یافته‌ها: قدرت عضله چهار سر ران (05/0 = P)، مسافت طي شده در آزمون لي لي تک پا (04/0 = P) و مدت انجام آزمون 8 (001/0 = P) پس از تکميل دوره مداخله بهبودي معني داري نشان دادند. بهبود گزارش فرد در پرسشنامه IKD و شاخص قرينگي اندام در آزمون لي لي تک پا از لحاظ آماري معني دار نبود. نتیجه‎گیری: به نظر مي‌رسد برنامه تمريني پيشنهادي بتواند اثر مطلوبي بر عملکرد بيماران مبتلا به آسيب ليگامنت متقاطع قدامي داشته باشد.

Title: بررسی شیوع اختلالات ستون فقرات در دانش‌آموزان با آسیب‌های بینایی و شنوایی

http://jrrs.mui.ac.ir/index.php/jrrs/article/view/1955

مقدمه: ستون فقرات، محور اصلی بدن است که نقش اصلي را در حفظ راستاي بدن ايفا مي‌کند. هدف از پژوهش حاضر بررسی شیوع ناهنجاری های ستون فقرات در دانش آموزان با آسیب بینایی و شنوایی است. مواد و روش‌ها: برای انجام پژوهش مطالعه از نوع مقطعی- توصیفی با نمونه‌گیری طبقه ای انتخاب شد. تعداد نمونه‌ها بر حسب ده درصد از دانش آموزان سال تحصیلی 91 انتخاب شدند که در شهر تهران 323 نفر، در شهرستان‌های استان تهران 244 نفر و در استان البرز 148 نفر بودند. از پرسشنامه دموگرافیک، شاقول، خط‌کش منعطف تایوانی و خط‌کش ساده برای جمع‌آوری داده‌ها استفاده شده وبااستفاده از آمار توصیفی گزارش شده است. یافته‌ها: دختران و پسران با آسیب بینایی استان البرز بیشترین میزان شیوع ناهنجاری اسکلیوز را دارند و در ناهنجاری کایفوز در چارك اول و سوم ، پسران با آسيب شنوايي استان البرز در شرايط بدتري بودند و در لوردوز در چارك اول دختران با آسيب شنوايي شهر تهران بيشترين مقدار و در چارك سوم پسران با آسيب بينايي استان البرز شرايط مشابهي داشتند. نتیجه‎گیری: بر اين اساس يافته‌ها مي‌توان نتيجه گرفت كه دانش آموزان استان البرز بيش از ساير دانش آموزان با آسيب‌هاي مشابه در شهر تهران و شهرستان‌هاي استان تهران دچار ناهنجاري‌هاي ستون فقرات هستند. بنابراین، نیاز است در این گروه‌ها، آموزش‌ها و نیز خدمات توانبخشی لازم جهت اصلاح ناهنجاری‌ها و یا در صورت لزوم استفاده  از حواس جایگزین در بدن ارائه شود.

Title: تأثیر شش هفته ماساژ درمانی بر افسردگی و کیفیت زندگی زنان افسرده

http://jrrs.mui.ac.ir/index.php/jrrs/article/view/1283

مقدمه: اختلالات خلقی از جمله افسردگی، زندگی بسیاری از مردم جهان را تحت تأثیر قرار می‌دهد. این بیماری به قدری شایع است که از آن به عنوان سرماخوردگی روانی یاد می‌شود. هدف این تحقیق مطالعه تأثیر شش هفته ماساژ درمانی بر میزان افسردگی و کیفیت زندگی زنان افسرده بود. مواد و روش‌ها: تعداد 20 بیمار زن افسرده که از پرسشنامه افسردگی Beck نمرات بالاتر از 18کسب نمودند، براساس نمرات افسردگی در دو گروه ماساژ درمانی (10بیمار با میانگین سنی 61/7 ± 20/31) وگروه شاهد (10 بیمار با میانگین سنی30/5 ± 20/27) قرار گرفتند. بیماران گروه ماساژ درمانی به مدت شش هفته و هر هفته سه جلسه ماساژ 30 دقیقه‌ای دریافت کردند در حالی که در مدت مشابه بیماران گروه شاهد به روند درمان خود ادامه دادند. برای اندازه‌گیری افسردگی و کیفیت زندگی بیماران به ترتیب از پرسشنامه افسردگی Beck و پرسشنامه Wisconsin قبل و بعد از اتمام دوره ماساژ استفاده شد. برای تجزیه و تحلیل داده‌ها از برنامه آماری SPSS و ANOVA Repeated measures و در سطح معناداری 05/0 استفاده شد. یافته‌ها: میزان افسردگی و کیفیت زندگی در پس آزمون گروه ماساژ درمانی به طور معنی‌داری متفاوت از مرحله پیش آزمون است یعنی اعمال مداخله موجب کاهش افسردگی و بهبود کیفیت زندگی بیماران شده‌ است (05/0P < ). نتیجه‎گیری: با توجه به نتایج به دست آمده از تحقیق حاضر، به نظر می‌رسد ماساژ درمانی در کاهش میزان افسردگی و کیفیت زندگی بیماران مؤثر است. بنابراین یافته‌های این پژوهش از تأثیر مثبت ماساژ درمانی بر بیماران افسرده حمایت می‌کند.

Title: تأثیر ریتم شبانه‌روزی بر تغییرات حس وضعیت مفصل زانو در فوتبالیست‌های مرد دانشگاهی

http://jrrs.mui.ac.ir/index.php/jrrs/article/view/1914

مقدمه: اختلال در عملکرد حس عمقی می‌تواند خطر بروز آسیب دیدگی در ورزشکاران را افزایش دهد. تاکنون تحقیقات متعددی عوامل تأثیرگذار بر روی دقت حس عمقی را مورد بررسی قرار داده است اما با این وجود تأثیر ریتم شبانه روزی بر عملکرد این حس مشخص نیست. بنابراین هدف از انجام این تحقیق بررسی تأثیر ریتم شبانه روزی بر دقت عملکرد حس عمقی مفصل زانو در مردان فوتبالیست دانشگاهی بود. مواد و روش‌ها: تعداد 30 فوتبالیست مرد دانشگاهی با میانگین سنی 27/1 ± 14/23 سال، قد 96/5 ± 2/176 سانتی متر و وزن 18/7 ± 94/69 بر اساس معیارهای ورود و خروج از تحقیق به عنوان آزمودنی انتخاب شدند. خطای بازسازی زاویه‌ای مفصل زانو در سه ساعت مختلف 10، 15 و 20 و در دو زاویه هدف 45 درجه و 60 درجه فلکشن توسط روش مارکرگذاری پوستی و عکس برداری دیجیتال اندازه گیری شد. برای تجزیه تحلیل عکس ها از نرم افزار اُتوکد و برای مقایسه داده‌ها در ساعات مختلف از Repeated measures ANOVA استفاده شد. یافته‌ها: با وجود ثبت رکوردهای متفاوت در ساعات مختلف و همچنین کمتر شدن میانگین خطاهای بازسازی در ساعات میانی روز، اختلاف معنی‌دار آماری در نتایج اندازه گیری‌های خطای بازسازی زاویه‌ای مفصل زانو در ساعات مختلف روز مشاهده نشد. نتیجه‎گیری: با توجه به نتایج به دست آمده از این تحقیق، به نظر می‌رسد که دقت عملکرد حس عمقی در مفصل زانو تحت تأثیر ریتم شبانه روزی قرار نداشته و بر این اساس، تفاوتی در احتمال بروز آسیب دیدگی ورزشکاران در ساعات مختلف شبانه روز ناشی از عملکرد متفاوت این حس وجود نداشته باشد.

Title: ساخت و بررسی ویژگی‌های روان‌سنجی آزمون دایکوتیک هجای بی‌معنی در بزرگسالان هنجار فارسی زبان

http://jrrs.mui.ac.ir/index.php/jrrs/article/view/2265

مقدمه: بازشناسی گفتار دایکوتیک شامل ارائه‌ همزمان محرکات رقابتی به گوش راست و چپ می‌باشد کهجهت بررسی برتری نیمکره‌های مغزی از نظر عملکرد زبانی و توانمندی‌های پردازش شنیداری در کودکان و بزرگسالان مورد استفاده قرار می‌گیرد. این مطالعه با هدف ساخت نسخه‌ فارسی آزمون دایکوتیک هجای بی‌معنی و بررسی ویژگی‌های روان‌سنجی آن انجام شده است. مواد و روش‌ها: مطالعه‌ حاضر از نوع ابزارسازی و غیر تجربی با الگوی متدولوژیکالاست که شامل سه بخش اصلی ساخت نسخه‌ فارسی آزمون دایکوتیک هجای بی‌معنی، بررسی روایی محتوا با استفاده از نسبت روایی محتوا و اجرای آن بر روی 80 فرد جوان و هنجار 18 تا 25 سال (40 مرد و 40 زن) با میانگین سنی 28/2 ± 16/ 21 سال به منظور بررسی اعتبار آزمون (ثبات درونی و آزمون- بازآزمون) می‌باشد، که با توجه به معیارهای ورود به مطالعه، از بین دانشجویان دانشگاه‌های تهران انتخاب شده بودند. یافته‌ها: نسبت روایی محتوا برای هر یک از گزینه‌های آزمون بالاتر از 62/0 بود. ضریب Cronbach's alpha و همبستگی بین‌رده‌ای به ترتیب 86/0 و 75/0 همبستگی آزمون- بازآزمون بعد از 2 تا 4 هفته، 83/0 محاسبه شد. نتیجه‎گیری: بر اساس یافته‌های پژوهش حاضر به نظر می‌رسد که نسخه‌ فارسی آزمون دایکوتیک هجای بی‌معنی تولید شده از روایی محتوا و اعتبار قابل قبول برخوردار بوده و می‌توان از آن در تحقیقات بالینی و پژوهشی استفاده نمود.

Title: مقایسه زمان فعالیت الکتریکی عضلات مرکزی تنه حین تکلیف فرود در افراد سالم و بیماران بعد از جراحی بازسازی رباط متقاطع قدامی

http://jrrs.mui.ac.ir/index.php/jrrs/article/view/2191

مقدمه: بسیاری از آسیب‌های رباط متقاطع قدامی زانو حین فعالیت‌های پرتنش مانند فرود ایجاد می‌شوند. فعالیت‌های پیش بینانه عضلات مرکزی تنه می‌توانند باعث کنترل فعالیت‌های پرتنش شوند و از ایجاد آسیب جلوگیری کنند. هدف این مطالعه، بررسی فعالیت الکتریکی پیش بینانه عضلات تنه در افراد سالم و بیماران بعد از جراحی بازسازی رباط متقاطع قدامی (Anterior cruciate ligament reconstruction یا ACLR) است. مواد و روش‌ها: در این مطالعه 11 فرد سالم و 11 فرد بیمار که تحت جراحی بازسازی لیگامان متقاطع قدامی قرار گرفته‌اند و بین 18-40 سال سن داشتند و 6 ماه تا 2 سال از زمان جراحیشان گذشته بود تحت مطالعه قرار گرفته‌اند. فعالیت الکتریکی عضلات گلوتئوس مدیوس، اکسترنال ابلیک ابدومینوس، کوادراتوس لومباروم و ارکتور اسپاین هر دو گروه حین تکلیف فرود ثبت شد. آزمون عملکردی پرش متقاطع و پرسشنامه International Knee Documentation Committee (IKDC) در هر دو گروه مورد سنجش قرار گرفت و با هم مقایسه شد. در همه تحلیل‌ها، سطح معنی داری کمتر از 05/0در نظر گرفته شد. یافته‌ها: یافته‌های مطالعه نشان داد که نمرات پرسشنامه و آزمون عملکردی در افراد سالم بطور معنی‌داری (پرسشنامه 001/0P ≤  و آزمون عملکردی039/0 P =) بیشتر از افراد بیمار بود. همچنین زمان بروز فعالیت فیدفورواردی عضلات گلوتئوس مدیوس (028/0 P = )،اکسترنال ابلیک ابدومینیس (030/0P = ) و ارکتور اسپاین (028/0 P = ) در افراد سالم بطور معنی‌داری زودتر از افراد بیمار بود .عضله کوادراتوس لومباروم (191/0P = ) تفاوت معنی‌داری نداشت. نتیجه‎گیری: در این تحقیق مشاهده شد که فعالیت الکتریکی و الگوی وارد عمل شدن عضلات تنه در افراد سالم و بیمارانی که تحت جراحی بازسازی لیگامان متقاطع قدامی قرار گرفته‌اند، متفاوت است. گروه بیماران هم فعالیت پیش بینانه خوبی نداشتند و هم کاهش سطح عملکرد و نمره پرسشنامه IKDC داشتند، که نشان دهنده مشکل در این افراد و نیاز به بازآموزی بیشتر است.

Title: تأثير برنامه جامع گرم كردن فیفا +11 در پيشگيري از آسيب‌هاي مچ پاي بازيكنان فوتبال: كارآزمايي باليني شاهددار تصادفي

http://jrrs.mui.ac.ir/index.php/jrrs/article/view/2280

مقدمه: اسپرین (Sprain) مچ پا شایع‌ترین آسیب بازیکنان فوتبال است. مطالعات قبلی نیز نشان داده‌اند که برنامه جامع گرم کردن فیفا +11 به‌طورکلی آسیب‌های ورزشی بازیکنان فوتبال را کاهش می‌دهد اما اطلاعات محدودی در مورد اثربخشی این برنامه بر پیشگیری از آسیب‌های مچ بازیکنان فوتبال وجود دارد؛ بنابراین هدف این کارآزمایی بالینی شاهددار تصادفی، بررسی اثر برنامه +11 بر آسیب‌های مچ بازیکنان فوتبال جوانان مرد ایران بود. مواد و روش‌ها: 980 بازیکن 15 تا 19 ساله مرد از 35 تیم رده سنی جوانان فوتبال ایران به‌صورت داوطلبانه حاضر به همکاری در این تحقیق شدند. این تیم‌ها به‌صورت تصادفی به دو گروه شاهد (17 تیم، 476 بازیکن) و مداخله (18 تیم، 504 بازیکن) تقسیم شدند. تیم‌هایی که در گروه مداخله قرار گرفتند حداقل دو بار در هفته در طول یک‌فصل، برنامه جامع فیفا +11 را به‌عنوان گرم کردن قبل از تمرین انجام می‌دادند اما بازیکنان گروه شاهد به انجام تمرینات عادی خود ادامه دادند. تعداد ساعات فعالیت بازیکنان گروه شاهد و مداخله در مسابقات و تمرین، به همراه آسیب‌های آن‌ها در طول مدت مطالعه به‌صورت آینده‌نگر توسط کادر پزشکی و مربیان ثبت می‌گردید. یافته‌ها: در طول 7 ماه مطالعه 67383 ساعت فعالیت بازیکنان و 1220 آسیب ثبت گردید. نتایج آزمون Poisson نشان داد که میزان بروز آسیب‌های مچ پای بازیکنان گروه مداخله (65/3 آسیب در هر هزار ساعت) در مقایسه با بازیکنان گروه شاهد (84/6 آسیب درهرهزار ساعت) کمتر بود (05/0 > P). نتیجه‎گیری: نتایج این مطالعه نشان داد تمرینات +11 می‌توانند از بروز آسیب‌های مچ پا پیشگیری نمایند بنابراین به بازیکنان، مربیان و بدن‌سازان فوتبال توصیه می‌گردد قبل از آغاز تمرین از این برنامه جامع گرم کردن استفاده نمایند.

Title: تاثیر دو روش تمرینات اصلاحی سنتی و تمرینات اصلاحی آکادمی ملی طب ورزش آمریکا (NASM) بر اصلاح عارضه لوردوزکمری دانشجویان دختر

http://jrrs.mui.ac.ir/index.php/jrrs/article/view/2279

مقدمه: هایپرلوردوزیس یکی از ناهنجاری‌های شایع در میان دختران می‌باشد و دستیابی به یک برنامه اصلاحی مناسب با هدف کاهش زاویه لوردوز کمری همواره مورد توجه بوده است، لذا هدف از این مطالعه مقایسه تأثیر دو روش تمرینات اصلاحی سنتی و تمرینات اصلاحی آکادمی ملی طب ورزش آمریکا (National Academy of Sports Medicine یا NASM) بر اصلاح عارضه لوردوزکمری دانشجویان دختر بود. مواد و روش‌ها: در این مطالعه نیمه تجربی تعداد 400 نفر از دانشجویان دختر 18 تا 22 ساله دانشگاه شهرکرد به عنوان نمونه اولیه با استفاد از روش نمونه‌گیری در دسترس انتخاب و مورد غربالگری قرار گرفتند. در پایان تعداد 45 نفر از آن‌ها که دارای زاویه انحنایی بیش از 30 درجه بودند انتخاب و به صورت تصادفی در سه گروه تمرینات سنتی، تمرینات  NASMو شاهد قرار گرفتند. گروه‌های تجربی به مدت 8 هفته، هفته‌ای 3 جلسه و هر جلسه 60 دقیقه در تمرینات شرکت کردند. قبل و بعد از مدخله میزان زاویه لوردوز کمری ارزیابی شد. به منظور تجزیه و تحلیل داده‌ها از آزمونANOVA  Repeated measure در سطح معنی‌داری 05/0 >P  استفاده شد. یافته‌ها: تعامل معنی‌داری در زاویه لوردوز کمری سه گروه و تست‌ها مشاهده شد (01/0 >P  و 3/43 = (42و2)F). به طوری که تفاوت بین گروه NASM نسب به گروه شاهد (9/0 =ES  و 01/0 >P  و 2/95 = (42 و 2)F) و تفاوت بین گروه سنتی نسبت به گروه شاهد معنی‌دار بود (7/ =ES و 01/0>P و2/17 = (42 و 2)F). نتیجه‎گیری: با توجه به نتایج بدست آمده از پژوهش حاضر، تمرینات NASM نسبت به تمرینات سنتی باعث کاهش بیشتری در انحنای قوس کمری شدند. بنابراین درمانگران و متخصصان حرکات اصلاحی می‌توانند از این تمرینات به عنوان یک روش تمرینی نوین جهت اصلاح ناهنجاریهای لوردوز کمری استفاده کنند.
